# Dermocosmetics in Acne Vulgaris: South African Consensus Recommendations With a Focus on Skin of Color

**DOI:** 10.1111/jocd.70696

**Published:** 2026-02-13

**Authors:** Willem I. Visser, Susanna M. Kannenberg, Alice Prevost, Nokubonga Khoza, Lushen Pillay, Kimberley J. Wiid, Izolda R. Heydenrych

**Affiliations:** ^1^ Division of Dermatology, Department of Medicine, Faculty of Medicine and Health Sciences Tygerberg Academic Hospital and Stellenbosch University Cape Town South Africa; ^2^ Dermatology House Cape Town South Africa; ^3^ Dr N Khoza, Dermatology and Laser Johannesburg South Africa; ^4^ Dermatology Department, Helen Joseph Hospital University of the Witwatersrand Johannesburg South Africa; ^5^ L'Oréal Dermatological Beauty Johannesburg South Africa; ^6^ Cape Town Cosmetic Dermatology Centre Cape Town South Africa

**Keywords:** acne vulgaris, acne‐induced hyperpigmentation, adjunctive therapy, dermocosmetics, skin of color

## Abstract

**Background:**

Acne vulgaris is one of the most common dermatological disorders worldwide, affecting both adolescents and adults. It frequently leads to significant psychosocial and physical sequelae, including acne‐induced hyperpigmentation and scarring. Beyond pharmaceutical therapies, dermocosmetics—topical formulations enriched with active ingredients in cosmetically elegant vehicles—have emerged as essential partners in acne management. They optimize clinical outcomes by supporting skin barrier repair, reducing irritation associated with conventional treatments, and targeting key pathogenic pathways in acne. However, no region‐specific guidance exists to inform the effective use of dermocosmetics in South African patients, particularly those with skin of color.

**Aims:**

To develop expert consensus recommendations for the use of dermocosmetics in acne vulgaris management within the South African context.

**Methods:**

This consensus was developed through structured expert meetings and a targeted literature review of current international and local evidence. The panel synthesized clinical experience and research findings to identify key principles for selecting and integrating dermocosmetics into acne treatment regimens.

**Results:**

The consensus highlights the multifaceted role of dermocosmetics in addressing core acne pathogenic factors, supporting epidermal barrier function, mitigating treatment‐related adverse effects, and managing acne‐induced hyperpigmentation in skin of color. Practical recommendations are provided for their use as adjunctive therapy, monotherapy in mild cases, and maintenance therapy to sustain remission.

**Conclusion:**

This South African consensus provides a practical, evidence‐informed framework for incorporating dermocosmetics into acne management, with particular attention to the needs of patients with skin of color. Adoption of these recommendations may enhance treatment outcomes, adherence, and patient satisfaction across diverse skin types.

## 1 Introduction

Acne vulgaris (AV) is a chronic inflammatory disorder of the pilosebaceous unit. It is one of the most ubiquitous dermatological conditions across the lifespan, affecting approximately 85% of adolescents and often persists into adulthood [1]. Clinical manifestations include lesions such as open and closed comedones, papules, pustules, nodules, and cysts, often leading to pigmentary changes and scarring [1]. This pigmentary sequela, termed acne‐induced hyperpigmentation (AIH), represents a subset of post‐inflammatory hyperpigmentation (PIH) specific to acne lesions [2]. Beyond physical morbidity, AV exerts profound psychological effects, impairing social interaction, self‐esteem, and reducing quality of life [3]. Pharmacological therapies, particularly topical and systemic retinoids, remain the mainstay of acne management but are frequently associated with side effects that may compromise the skin barrier, induce irritation, and reduce adherence [4]. Dermocosmetics (DCs) combine dermatologically active ingredients with cosmetically elegant formulations, providing an integrative approach to acne management. DCs not only address treatment‐related side effects but also offer synergistic therapeutic benefits targeting key pathogenic mechanisms [5, 6, 7]. As the skincare market continues to expand and diversify, a thorough understanding of available formulations, active ingredients, and mechanisms of action is essential for optimal patient care, underscoring the need for structured, evidence‐based education on DC selection for both patients and healthcare providers.

### 1.1 Aims

This consensus statement aims to clarify the role of DCs in acne vulgaris AV management, with particular emphasis on patients with skin of color (SOC), in whom AIH is often the most distressing and persistent outcome. This consensus statement provides a practical, evidence‐based framework to optimize clinical outcomes, minimize disease burden, and integrate early preventive strategies that reduce scarring and pigmentation complications.

## 2 Methodology

A panel of six dermatologists with extensive experience in acne management and DC therapies convened to develop this consensus statement. The process involved a structured literature review and three expert meetings held over 3 months.

### 2.1 Literature Review

A targeted search of the Medline and PubMed databases was performed using the terms ‘acne vulgaris’ and ‘dermocosmetics’. The search focused on clinical trials, meta‐analyses, and review articles published within the last decade. Results were restricted to English‐language publications. Key areas of focus included the efficacy and safety of DCs in acne treatment, their use as monotherapy or adjunctive therapy, and their impact on AIH.

### 2.2 Consensus Process

Three formal meetings were held to review the literature, discuss key active ingredients and mechanisms of action, evaluate clinical applications of DCs across diverse patient populations, and develop practical recommendations. Consensus was achieved through structured discussion and unanimous agreement among all panel members. No formal voting procedures were required.

### 2.3 Consensus Statement Development

After the final meeting, the draft consensus statement was circulated among all participants for review and approval. The finalized statement reflects a synthesis of the best available evidence and expert clinical judgment, providing practical guidance for integrating DCs into acne management strategies.

## 3 Results

### 3.1 Definition and Rationale for the Use of Dermocosmetics in Acne Management

DCs are topical formulations that combine active ingredients targeting specific dermatological pathways with cosmetically elegant vehicles designed for regular use [6]. While many DCs undergo clinical evaluation, clinical testing is not a defining requirement [6]. Their development focuses on addressing pathogenic mechanisms, supporting epidermal barrier function, providing hydration, offering photoprotection, and improving tolerability, particularly during concomitant pharmacologic therapy [6].

In AV management, DCs act as valuable adjuncts to conventional therapies by acting on core pathogenic factors, such as excess sebum production, abnormal follicular keratinisation, microbial dysbiosis, and inflammation [6]. Some formulations also include depigmenting agents to reduce AIH, a concern that is especially relevant in patients with SOC [8, 9].

DCs can therefore be used both as adjunctive therapy to enhance efficacy and tolerability of pharmacological regimens, and as monotherapy in mild or maintenance phases of acne, supporting long‐term control and overall skin health.

### 3.2 Dermocosmetics as Monotherapy in Acne Vulgaris Management

AV is a multifactorial disease characterized by four key pathogenic processes: excess sebum production, abnormal keratinisation, proliferation and dysbiosis of *Cutibacterium acnes*, and inflammation [5, 10].

DCs, when used as monotherapy, are formulated to target these pathways, providing therapeutic benefits with a lower risk of side effects compared to pharmacological treatments [5, 6, 11]. Their use is particularly supported in mild acne and in specific populations where conventional therapies may be contraindicated or limited, such as pregnant women, children under 10 years, and patients intolerant or sensitive to topical or systemic medication [5].

**FIGURE 1:**
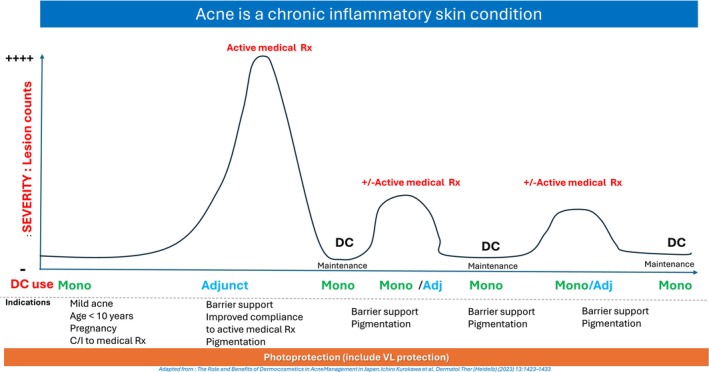
Conceptual model illustrating the role of dermocosmetics across acne management phases (adapted from Kurokawa I et al. [7]).

Given that the course of AV often spans decades, DCs play a valuable role in maintenance therapy between active treatment phases, providing a well‐tolerated option throughout the lifespan of the condition (Figure 1) [7]. However, it is important to acknowledge the limitations of DCs in treating more severe cases of AV. Their use should not delay the timely initiation of prescription therapies where clinically indicated. Table 1 summarizes key active ingredients found in DCs and their targeted actions on acne pathogenic pathways.

**TABLE 1:** Categories of dermocosmetic ingredients and their targeted actions in acne pathophysiology (adapted from Thiboutot et al. [5]).

Dermocosmetic ingredients for acne					
Keratolytics	Anti‐inflammatory	Sebum control	Antibacterial	Barrier/microbiome	Photoprotection
Alpha‐hydroxy acid	Azeleic Acid	Bakuchiol	AMP	Azeleic Acid	Broad spectrum
Azeleic Acid	Bakuchiol	Bixa Orellana seed extracts	Bakuchiol	Ceramides	SPF 50+
Gluconolactone	Bixa Orellana seed extracts	EGCG	BPO	Procerad	**Organic filters:**
Glycolic acid	Decanediol	Niacinamide	Decanediol	Glycerin	**UVB filters:**
HEPES	Gallic acid	Zinc	Gallic acid	Mannose	Octinoxate (EHMC)
Linoleic acid	Lichocalcone A	Phylobioma	Lactobacillus plantarum	Niacinamide	Octocrylene
Lipohydroxy acid	Niacinamide		Lauric acid	Panthenol	Homosalate
Papain	Panthenol		Mannose	Shea butter	Ensulizole
Phylobioma	Phylobioma		Piroctone olamine	Phylobioma	**UVA filters:**
Retinol derivatives	Salix alba		Phylobioma	Vitreoscilla filiformis (APF)	Avobenzone
Salicylic acid	Soy isoflavone		Vitreoscilla filiformis (APF)	Zinc	Mexoryl SX (ecamsule)
Sylibium marianum	Sylibum marianum		Zinc		Mexoryl XL
	Zinc				Mexoryl 400
					Tinosorb S
					Tinosorb A2B
					Tinosorb M
					**Inorganic (mineral) UV filters**
					Titanium dioxide (micronized vs non‐micronized)
					Zinc oxide (micronized vs non‐micronized)
					**HEVL protection**
					Iron oxide
					Titanium dioxide (non‐micronized)
					Zn oxide (non‐micronsed)
					TriAsorB

#### 3.2.1 Sebum Production

Excessive sebum production, driven by androgenic stimulation of sebaceous glands, fosters proliferation and dysbiosis of *C. acnes* and promotes comedogenesis through follicular hyperkeratinisation [5, 10]. Emerging evidence also suggests that AV is associated with inherent interfollicular epidermal barrier dysfunction linked to the lipid‐rich, oily environment [10, 12].

DC ingredients such as niacinamide, L‐carnitine, and green tea extracts have demonstrated sebostatic properties [5]. Niacinamide, in particular, reduces sebum production while also exerting anti‐inflammatory effects. Fullerene and epigallocatechin‐3‐gallate (EGCG) from green tea offer additional antioxidant and sebostatic benefits, making them valuable in managing mild acne.

#### 3.2.2 Abnormal Keratinisation

Follicular hyperkeratinisation, characterized by impaired shedding of keratinocytes within the pilosebaceous unit, initiates the formation of microcomedones that evolve into visible lesions [4, 5].

Keratolytic agents commonly found in DCs, such as salicylic acid (SA), lipo‐hydroxy acid (LHA), alpha‐hydroxy acids (AHAs), and retinol derivatives normalize desquamation and help prevent comedonal acne.

#### 3.2.3 *C. acnes* Proliferation and Dysbiosis

The proliferation of *C. acnes* within occluded follicles exacerbates acne through biofilm formation, enhanced virulence, and activation of Th17‐mediated inflammatory pathways [10, 13]. Reduced microbial diversity, particularly enrichment of phylotype IA1, is associated with increased disease severity [10].

DC ingredients such as niacinamide, zinc salts, and antimicrobial agents like *Salix alba* (white willow extract), combined with 1,2‐decanediol, help regulate the skin microbiome and reduce *C. acnes* colonization, offering a strategy to minimize reliance on systemic antibiotics and mitigate resistance development.

Additionally, prebiotics, probiotics, and postbiotics have shown promise in supporting microbial diversity, suppressing *C. acnes* overgrowth, and modulating local inflammatory responses. Their inclusion in DC formulations may offer adjunctive benefits in microbiome‐targeted acne therapy [14, 15].

#### 3.2.4 Inflammation

Inflammation is central to acne lesion progression, driven by innate and adaptive immune responses to microbial and barrier disruption [5, 10]. *C. acnes* induce cytokine release, promoting inflammatory cascades.

Anti‐inflammatory DC ingredients, including niacinamide, alpha‐linolenic acid, zinc salts, and botanical extracts such as soy isoflavones, help reduce oxidative stress and downregulate pro‐inflammatory mediators.

### 3.3 Dermocosmetics as Adjunctive Therapy in Acne Vulgaris Management

AV is increasingly recognized not only as an inflammatory disorder but also as a condition associated with skin barrier dysfunction, characterized by reduced levels of essential lipids such as ceramides [10, 16]. Impaired ceramide content leads to increased transepidermal water loss (TEWL), resulting in heightened susceptibility to irritation, inflammation, and secondary dysbiosis [10, 16, 17]. Dysbiosis, particularly the proliferation of *C. acnes* phylotype IA1, further contributes to inflammatory processes and barrier disruption [10].

The importance of preserving barrier integrity and maintaining a healthy microbiome in acne management cannot be overemphasized [10]. However, conventional acne therapies—including topical retinoids, benzoyl peroxide, and systemic isotretinoin—commonly exacerbate barrier dysfunction, causing dryness, scaling, and irritation [4, 5].

Appropriately formulated DCs, particularly gentle cleansers and moisturizers, play a pivotal role in adjunctive therapy.

They support epidermal barrier restoration, maintain microbial balance, and mitigate treatment‐induced side effects, thereby improving overall therapeutic adherence [5, 18, 19]. We have proposed an ideal three‐step DC skincare routine for AV patients:

### 3.4 Cleansers

Mild, pH‐balanced cleansers are essential for acne patients undergoing treatment. These formulations effectively remove excess sebum, debris, and makeup without compromising the stratum corneum or altering the skin's physiological pH. Harsh surfactants can strip the skin of natural lipids, worsening dryness and irritation, and ultimately reducing adherence to prescribed therapies. Optimal cleansers for acne‐prone skin should be non‐comedogenic, maintain an acidic pH (~5.5), and be free from aggressive detergents [6].

### 3.5 Moisturizers

Moisturizers containing barrier‐repairing ingredients, particularly ceramides, hyaluronic acid, and essential fatty acids, replenish depleted extracellular lipids, reduce TEWL, and enhance barrier function [6]. Ceramides restore the lipid matrix of the stratum corneum, and hyaluronic acid, a component of the natural moisturizing factor, enhances hydration while remaining non‐comedogenic [6].

### 3.6 Photoprotection

Photoprotection is an essential adjunct in acne management, not only to prevent ultraviolet B (UVB)–induced erythema but also to mitigate photoaging, dyspigmentation, DNA damage, and photocarcinogenesis [20, 21]. This is particularly relevant as many conventional acne treatments, including topical and systemic retinoids and tetracycline antibiotics, enhance photosensitivity [20]. UVA contributes to dermal matrix degradation, while UVB promotes keratinocyte proliferation and sebum production, aggravating acne‐prone skin [20].

In individuals with SOC, visible light (VL), particularly in the blue spectrum (400–500 nm), plays a critical role in inducing or worsening hyperpigmentation [20, 21]. Therefore, effective photoprotection in these patients should extend beyond the UV range to include VL. Standard organic UV filters have limited efficacy against VL, but protection can be achieved through the inclusion of non‐micronized inorganic particles (zinc oxide, titanium dioxide), iron oxides, pigmentary titanium dioxides, and newer organic molecules that absorb within the visible spectrum [20].

However, cosmetic elegance remains a key determinant of adherence. Many tinted formulations still leave a white or gray residue, limiting acceptance among patients with darker skin tones. This underscores the need for more inclusive, shade‐matched formulations that provide both effective protection and aesthetic acceptability. Clinicians should advocate for such advances and help patients identify the most cosmetically suitable products currently available [22].

Sunscreens for acne‐prone skin should offer broad‐spectrum UVA/UVB protection, with SPF ≥ 50, light, water‐based, non‐comedogenic, fast‐absorbing, and mattifying formulations. Emerging evidence supports the use of sunscreens containing photolyases (DNA repair enzymes) and suggests potential benefits of oral and topical antioxidants in protecting against visible light–induced pigmentation [23, 24].

### 3.7 Acne‐Induced Hyperpigmentation (AIH)

AIH is a frequent and distressing sequela of acne, particularly among individuals with darker skin tones [8, 9]. Management begins with effective control of active acne to prevent further pigmentary change [8, 9].

Photoprotection remains the cornerstone of PIH prevention and treatment, given the significant role of ultraviolet (UV) and visible light exposure in exacerbating pigmentation [20, 21].

In addition to prescription depigmenting agents, numerous DC formulations containing brightening actives—such as Melasyl, Thiamidol, niacinamide, vitamin C, liquorice extract, and tranexamic acid—are widely used to improve post‐inflammatory hyperpigmentation, especially in SOC [25]. A full discussion of skin‐lightening or depigmenting DC lies beyond the scope of this consensus statement, but their potential contribution within the context of acne care and AIH is acknowledged.

A holistic, patient‐specific approach that combines ongoing acne control, daily broad‐spectrum photoprotection, and targeted depigmenting therapies is essential for achieving optimal outcomes in the management of AIH, particularly in patients with SOC [8, 9].

### 3.8 Practical Considerations for the Use of Dermocosmetics in Acne Management

The selection of DC products should be individualized based on the patient's clinical profile, including acne severity, skin type (e.g., oily, dry, or combination), underlying conditions such as sensitivity, rosacea, atopic dermatitis, or eczema, and any concurrent pharmacologic treatments [5, 6].

Age‐specific considerations are also important. Adolescents typically have more sebaceous activity and may benefit from lighter, mattifying moisturizers and oil‐control formulations. In contrast, adult acne patients, especially women, often present with combination or dry, sensitive skin and may require more hydrating, barrier‐restoring products containing ceramides and hyaluronic acid [26].

Acne cosmetica—particularly pomade acne—is frequently encountered in younger individuals with SOC. The use of heavy oils, pomades, and other comedogenic haircare products can lead to persistent comedonal and inflammatory lesions, especially on the forehead and temporal regions. These presentations may be resistant to standard therapies unless cosmetic triggers are identified. A targeted clinical history and appropriate patient education are essential, and DCs for this population should be explicitly non‐comedogenic [27].

In cases of acne mechanica or ‘maskne’—an increasingly common form of frictional acne induced by prolonged mask use—barrier impairment is a key driver of lesion development. DCs containing ceramides, humectants, and anti‐inflammatory agents can help restore skin integrity, reduce irritation, and improve adherence to mask use during treatment [28].

Furthermore, unsupervised or prolonged use of topical corticosteroids, often for skin lightening or non‐specific dermatitis, can lead to steroid‐induced acneiform eruptions or exacerbate barrier dysfunction, particularly in patients with SOC. During corticosteroid withdrawal phases, these patients may exhibit increased sensitivity to standard acne therapies. In such scenarios, DCs that support epidermal barrier repair and provide gentle moisturisation are critical to reduce irritation, improve tolerance, and maintain skin integrity [29].

Dermatologists should familiarize themselves with high‐quality, evidence‐based DC formulations available in their market. Given the overwhelming number of products and marketing claims, patients often look to healthcare professionals for practical, individualized guidance. Offering branded recommendations, mindful of efficacy, tolerability, cosmetic elegance, and cost‐effectiveness, can simplify decision‐making for patients and improve adherence. This is particularly important in resource‐limited settings, where access and affordability are key determinants of treatment success [30].

### 3.9 The Role of Cosmetic Counseling in Acne Management

Effective acne management requires more than pharmacological prescriptions. Cosmetic counseling—offering structured guidance on DC selection, skincare routines, makeup use, and hair and scalp products—is increasingly recognized as a critical component of holistic acne care [5].

Patients with SOC may face unique challenges, including a higher risk of pigmentary sequelae, culturally specific grooming practices (e.g., use of heavy hair oils or pomades), and difficulty accessing cosmetically acceptable photoprotection or camouflage options [2]. Dermatologists play a key role in addressing these needs. This includes recommending non‐comedogenic, tinted sunscreens suited to deeper skin tones; advising on makeup choices that conceal acne or pigmentation without being comedogenic; identifying haircare products that may contribute to forehead acne; and supporting barrier repair and treatment adherence through the use of evidence‐based DCs.

By expanding the acne consultation to include these elements, clinicians can improve clinical outcomes, patient satisfaction, and long‐term disease control, particularly in populations historically underrepresented in dermatologic research and product development.

## 4 Conclusion

AV is a highly prevalent dermatological condition affecting individuals across a broad age range, often resulting in visible sequelae and profound psychological distress. The emergence of DCs has expanded the therapeutic landscape of acne management, providing complementary benefits to traditional pharmacological treatments.

DC formulations target key pathogenic pathways of acne, including excess sebum production, abnormal follicular keratinisation, microbial dysbiosis, and inflammation, while supporting epidermal barrier repair, hydration, and photoprotection. Importantly, DCs also help prevent and reduce AIH, a major concern among patients with SOC, where pigmentary disorders are more frequent and persistent. By improving tolerance to medical treatments and reducing irritation, DCs enhance adherence and patient satisfaction.

This consensus emphasizes the integration of DCs throughout acne treatment regimens: advocating for their use as effective monotherapy for mild acne, maintenance therapy between pharmacologic courses, and as adjunctive therapy in moderate to severe cases.

In patients with SOC, formulations that protect against visible light, promote pigmentary evenness, and maintain barrier integrity are particularly valuable. Cosmetic counseling should also form part of holistic acne care, incorporating guidance on DC use, makeup, and culturally relevant haircare practices. A personalized approach to DC selection and use can optimize clinical outcomes, improve quality of life, and address the unique needs of diverse patient populations.

## Author Contributions

W.I.V. and K.J.W. conceived the review and design. All authors searched the literature for relevant articles. The first draft of the manuscript was written by W.I.V. All authors critically reviewed, revised, and approved the final version of the manuscript.

## Funding

This work was supported by La Roche Posay, South Africa sponsored and facilitated the consensus meetings. The authors did not receive any payments or honoraria for the development of this article.

## Disclosure

*AI‐Assisted Technologies Disclosure*: During the preparation of this manuscript, the authors used ChatGPT (version 5.2, OpenAI) to support language editing and improve clarity and structure in selected sections. The tool was not used for generating scientific content, data analysis, or interpretation of evidence. All content was reviewed and approved by the authors, who take full responsibility for the integrity and accuracy of the manuscript.

## Ethics Statement

This article is based on previously published research and does not include any new studies involving human participants or animals. This work complies with the principles of the Declaration of Helsinki (1964) and its subsequent amendments.

## Conflicts of Interest

W.I.V. has previously received honoraria and served on advisory boards for Beiersdorf, NAOS, L'Oréal, Galderma, Genop Healthcare, ISDIN, and Pierre Fabre. K.J.W. is the Medical Director for L'Oréal South Africa. S.M.K., N.K., L.P., A.P., and I.R.H. declare no conflicts of interest. The meetings and discussions related to this publication were facilitated by L'Oréal Dermatological Beauty South Africa. However, all data interpretation and conclusions were independently formulated by the participating healthcare practitioners. K.J.W., in her capacity as Medical Director at L'Oréal Dermatological Beauty South Africa, had no influence over the conclusions presented and contributed only to the conceptualization and literature review elements of the manuscript.

## Data Availability

The data that support the findings of this study are available on request from the corresponding author. The data are not publicly available due to privacy or ethical restrictions.
